# Acoustic analog computing system based on labyrinthine metasurfaces

**DOI:** 10.1038/s41598-018-27741-2

**Published:** 2018-07-04

**Authors:** Shuyu Zuo, Qi Wei, Ye Tian, Ying Cheng, Xiaojun Liu

**Affiliations:** 1 Key Laboratory of Modern Acoustics, Department of Physics and Collaborative Innovation Center of Advanced Microstructures, Nanjing University, Nanjing, 210093 China; 2 Jiangsu Key Laboratory on Opto-Electronic Technology, School of Physics and Technology, Nanjing Normal University, Nanjing, 210023 China; 3 State Key Laboratory of Acoustics, Institute of Acoustics, Chinese Academy of Sciences, Beijing, 100190 China

## Abstract

Acoustic computing devices, including switches, logic gates, differentiator and integrator, have attracted extensive attentions in both academic research and engineering. However, no scheme of acoustic computing device with more complex functionality has been proposed, such as ordinary differential equation (ODE) solver. Here, we propose an acoustic analog computing (AAC) system based on three cascaded metasurfaces to solve the *n*th-order ODEs. The metasurfaces are constructed with layered labyrinthine units featuring broad amplitude and phase modulation ranges. The simulated transmitted pressure of the AAC system agrees well with the theoretical solution of ODE, demonstrating the excellent functionality. Unlike the optical ODE solver based on differentiator or integrator, whose geometry becomes more complicated for solving higher order ODE, the proposed AAC system with fixed geometry can be designed for arbitrary *n*th-order ODE in principle. The proposal may find applications in various scenarios such as acoustic communication, analog computing and signal processing.

## Introduction

Wave-based computing, which is considered as an alternative future computing due to the benefits of high-efficiency, low crosstalk and parallel processing, has attracted extensive attention in both academic research and engineering field. Analogous to the electronic counterparts, many computing devices, including integrators^1^ and differentiators^2^ have been proposed in optical domain. With the assistance of these basic units, the computing devices can be realized with more complicated optical functionalities such as optical ordinary differential equation (ODE)^3,4^. ODEs govern a wide variety of fundamental physical phenomena and engineering systems, and describe the classical models in the theory of signals and systems^5–7^. In acoustic domain, solving the ODE would revolutionize many application fields ranging from acoustic communication, analog computing to signal processing. However, no acoustic ODE solver has been proposed, and the traditional optical design scheme involving differentiator or integrator suffers from the complicated geometry for high-order ODEs. Meanwhile, the acoustic computing devices, such as diodes^8^, switches^9^ and logic gates^10,11^, have been demonstrated based on phononic crystals or bulk metamaterials, which suffer from the limitations of simple functionality, high losses, and geometrical complexity. Therefore, it is necessary for seeking a novel approach to design acoustic ODE solver which features fixed geometry and high efficiency.

As an exciting platform for manipulating acoustic waves with a sub-wavelength scale, acoustic metasurface may promise an alternative approach to design acoustic ODE solver. By strongly interacting with the incident waves, acoustic metasurfaces can effectively manipulate the amplitude and phase of acoustic waves^12–20^. As a result, numerous extraordinary phenomena and functionalities have been realized, such as perfect absorbing^12,13^, cloaking^14,15^, anomalous reflection/refraction^16,17^, holography^18,19^, and ultra-sparse reflection^20^. Recently, the concept of computational metasurface has been proposed to perform mathematical operations, such as spatial differentiation, integration and convolution^21,22^. However, these realized operations are relatively simple, which can only meet the basic requirements of acoustic signal processing. Our purpose is designing a system based on metasurface to solve ODEs, which is meaningful for the development of acoustic signal processing.

In this paper, we propose and numerically demonstrate an acoustic analog computing (AAC) system to solve the *n*th-order ODEs. The AAC system consists of three cascaded metasurfaces, i.e., two focusing metasurfaces (FMs) and one space filtering metasurface (SFM). The FM can perform the Fourier transform (FT), while the SFM exhibits complex phase and amplitude responses to function as a spatial filter. Both the FM and SFM are constructed by layered labyrinthine units, which can provide nearly complete modulations on phase and amplitude of the incident acoustic signals^23,24^. Numerical full-wave simulations show that the transmitted pressure of the AAC system agrees well with the theoretical solution of the ODE, confirming the excellent functionality of the acoustic AAC system.

## Results

### Prototype for solving ODEs based on the spatial Fourier transform

**Figure 1:**
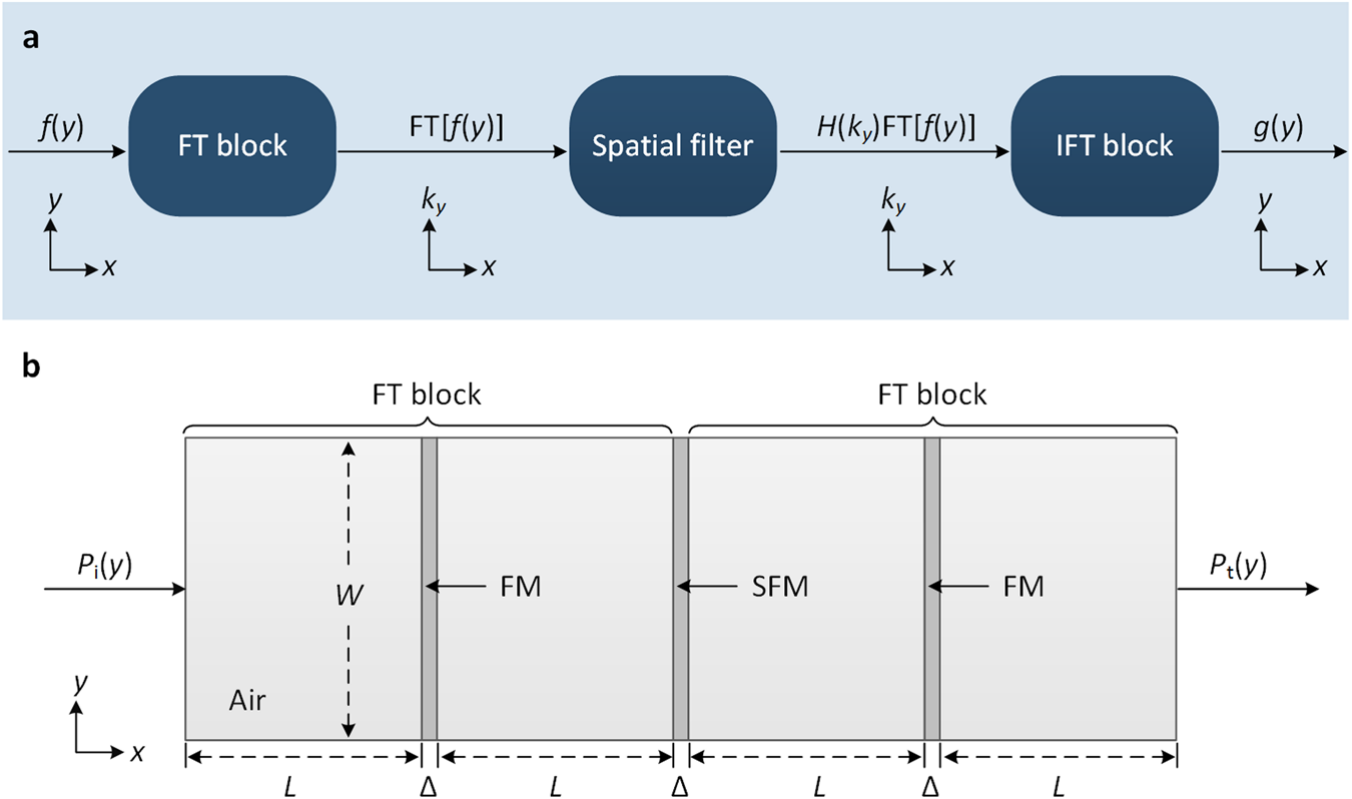
Concept to design the acoustic AAC system. (**a**) Sketch of solving ODEs based on the general concept of spatial FT. (**b**) Schematic diagram of the acoustic AAC system. The system consists of two FMs and one SFM. The width *W* and thickness Δ of the FM and SFM are 0.972 m and 0.04 m, respectively. The focal length of FM is *L* = 0.5488 m. Each FM and two neighboring blocks of background medium (air) form an acoustic FT block. The SFM with complex transmission coefficient functions as the spatial filter.

The common constant coefficient *n*th-order ODE is defined as

1 $${a}_{n}{g}^{(n)}(y)+{a}_{n-1}{g}^{(n-1)}(y)+\mathrm{...}+{a}_{1}g^{\prime} (y)+{a}_{0}g(y)=f(y)$$

where *f*(*y*) denotes the input function, *g*(*y*) represents the corresponding output function (equation solution), *g*^(*n*)^(*y*) is the *n*th-order derivative of *g*(*y*) (*n* ≥ 0), and *a*_*n*_ is the constant coefficient. Imposing the FT and the inverse FT (IFT), the equation solution can be expressed as

2 $$g(y)={\rm{I}}{\rm{F}}{\rm{T}}\{H({k}_{y}){\rm{F}}{\rm{T}}[f(y)]\}$$

with the transfer function being

3 $$H({k}_{y})={[{a}_{n}{(i{k}_{y})}^{n}+{a}_{n-1}{(i{k}_{y})}^{n-1}+\mathrm{...}+{a}_{1}(i{k}_{y})+{a}_{0}]}^{-1}$$

where *k*_*y*_ is the spatial frequency. Figure 1a shows a linear space-invariant system, which can realize the solving process described by Eq. (2)^25–27^. In this figure, the input function propagates along the *x* direction, the FT block performs the spatial FT on the input function, the spatial filter applies the transfer function *H*(*k*_*y*_), and the IFT block performs the spatial IFT. Therefore, the equation solution can be obtained by using FT and IFT blocks, along with the appropriate spatial filter.

### Design of the acoustic ODE solver

To solve ODEs in acoustics, we design the AAC system as shown in Fig. 1b, where *P*_i_(*y*) and *P*_t_(*y*) are incident and transmitted signals, respectively. Analogous to the system in Fig. 1a, the AAC system consists of three sub-blocks: (i) a FT block, (ii) a well-designed SFM, and (iii) a FT block. The FT block is achieved by using a FM [with the transmission coefficient *T*_f_(*y*)] together with two neighboring blocks of background medium^28^. The SFM with the transmission coefficient *T*_s_(*y*) can function as the spatial filter. The IFT block is not considered here, since it requires the negative metamaterial^21^. Regarding these facts, the transmitted signal of the AAC system can be described as

4 $${P}_{t}(y)={\rm{F}}{\rm{T}}\{{T}_{s}(y){\rm{FT}}[{P}_{i}(y)]\}$$

It is evident that Eq. (4) can be related mathematically to Eq. (2), provided that the incident signal *P*_i_(*y*), the real-space coordinate *y* at the SFM, and the transmission coefficient *T*_s_(*y*) are interpreted as the input function *f*(*y*), the spatial frequency *k*_*y*_, and the transfer function *H*(*k*_*y*_), respectively. The transmitted signal *P*_t_(*y*) is thus proportional to the mirror image of the desired solution *g*(*y*) owing to the relation of $g(\,-\,y)\propto {\rm{FT}}\{{\rm{FT}}[g(y)]\}$. As a result, the solution of the ODE can be obtained by appropriate tailoring of the transmission coefficient *T*_s_(*y*) to mimic the transfer function *H*(*k*_*y*_). Unlike the optical ODE solver based on differentiator or integrator, whose geometry becomes more complicated for higher order ODE^29^, the proposed AAC system with a fixed geometry is capable for arbitrary order ODE. Besides, the AAC system can perform mathematical operations by designing the transfer function, whose functionality contains and beyond the scope of the previous analog system^21,22^.

**Figure 2:**
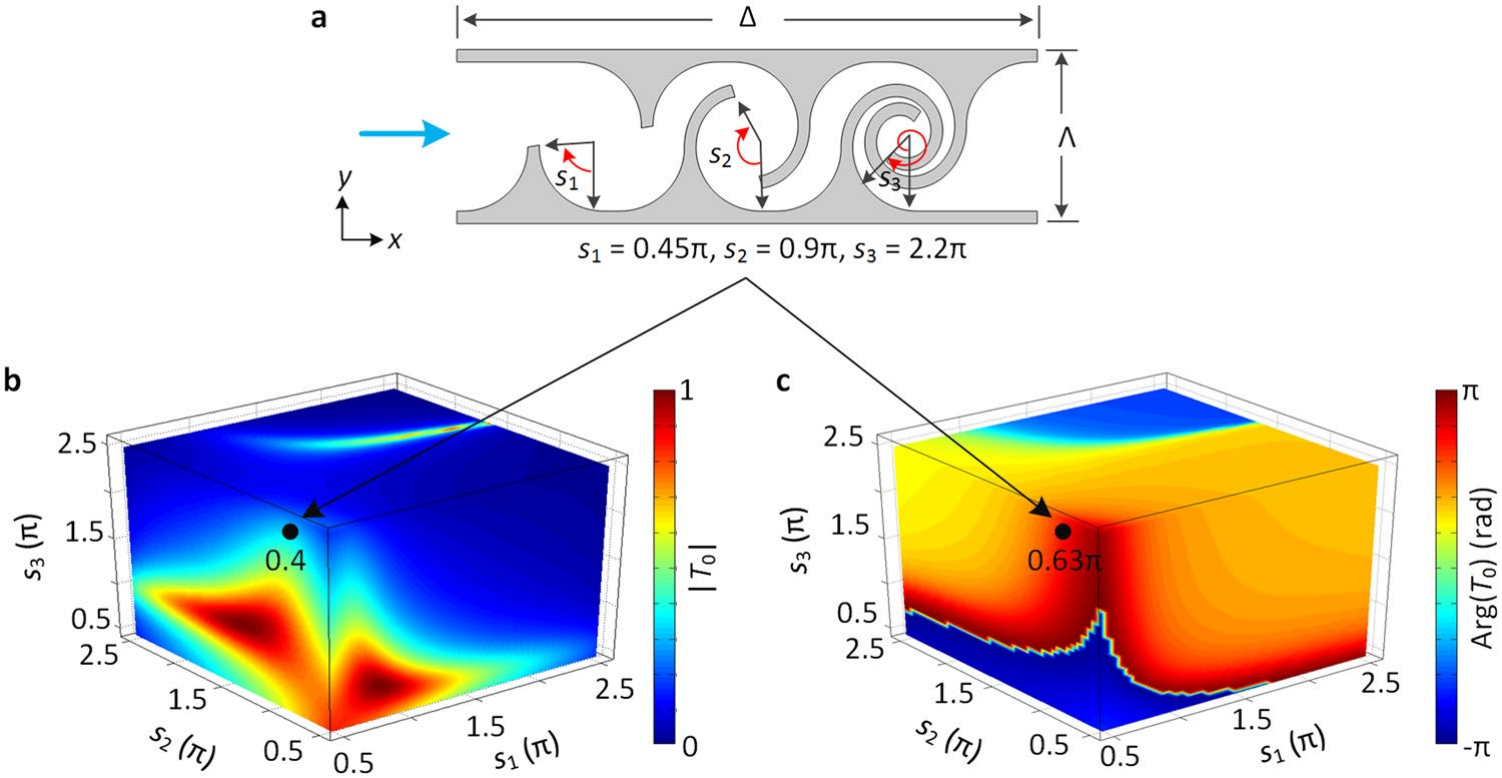
Optimize the layered labyrinthine unit. (**a**) Schematic diagram of the layered labyrinthine unit. The unit is composed of three tapered labyrinthine components with spiral radians *s*_1_, *s*_2_ and *s*_3_, respectively. Here, spiral radians are *s*_1_ = 0.45π, *s*_2_ = 0.9π and *s*_3_ = 2.2π. (**b**) Amplitude and (**c**) phase of the transmission coefficient ${T}_{0}({s}_{1},{s}_{2},{s}_{3})$ versus spiral radians *s*_1_, *s*_2_ and *s*_3_.

To construct the FM and the SFM, Fig. 2a shows the layered labyrinthine unit with a width Λ = 1.2 cm and a thickness Δ = 4 cm. The operating frequency is chosen as 2500 Hz, and the background medium is chosen as air (with a density $\rho =1.21\,{\rm{kg}}/{{\rm{m}}}^{{\rm{3}}}$ and a bulk modulus $\kappa =142.36\,{\rm{kPa}}$). The labyrinthine unit consists of three tapered labyrinthine components characterized by spiral radians *s*_1_, *s*_2_ and *s*_3_, respectively. The three spiral radians can independently vary from 0.45π to 2.55π. For an incident signal (blue arrow), the complex path-coiling of the labyrinthine unit and the induced impedance mismatch enhance the multi-scattering process, which results in the transmitted signal with tunable amplitude and phase^24^. The behavior allows the labyrinthine unit to function as an acoustic signal modulator that features broad amplitude and phase modulation ranges. Due to the property of broad modulation, the layered labyrinthine units have been used to perform mathematical operations^22^.

To demonstrate such a broad tuning, we numerical calculated the transmission coefficient ${T}_{0}({s}_{1},{s}_{2},{s}_{3})$. The calculated transmission amplitude and phase with various spiral radians *s*_1_, *s*_2_ and *s*_3_ are shown in three-dimensional color Fig. 2b and c, respectively. In Fig. 2(b,c), one coordinate point denotes one labyrinthine unit, and the value of the point denotes the transmission amplitude (or phase) of the corresponding unit. For example, to the sample unit in Fig. 2a, the transmission amplitude is 0.4 as shown by the black point in Fig. 2b, while the transmission phase is 0.63π as shown by the black point in Fig. 2c. Accordingly, the transmission coefficient of the sample unit is 0.4exp(0.63π*i*). In Fig. 2b and c, we can obtain a lot of labyrinthine units, whose transmission amplitudes range from 0 to 1, and transmission phases cover the entire 2π range. From these units, we can choose the desired units by minimizing $|T(m{\rm{\Lambda }})-{T}_{0}({s}_{1},{s}_{2},{s}_{3})|$. Here, −40 ≤ *m* ≤ 40 is an integer, *T*(*m*Λ) is the transmission coefficient of FM or SFM. By assembling the 81 chosen units in the *y* direction, we can construct the FM or SFM. Therefore, the FM and SFM with the complex transmission responses can be constructed with the labyrinthine units by elaborately tailoring the spiral radians. It should be noted that the spiral radian is not a periodic function, and the range of *T*_0_ depends on the range of spiral radian (Fig. 2b and c). The spiral radian is thus expected to have a wide range. Nevertheless, when the spiral radian is above 2.55π, the two spiral fins will touch together, prohibiting the propagation of signal. Besides, when the spiral radian is below 0.45π, the labyrinthine component can be considered as a waveguide, whose manipulate ability is very weak. Therefore, the range of spiral radian is set as 0.45π to 2.55π.

### Construct the FT block

**Figure 3:**
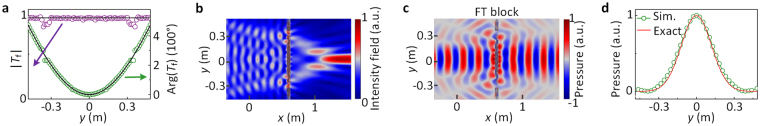
Acoustic FT block. (**a**) Designed discrete transmission coefficient (circles) and the required ideal transmission coefficient (lines) of FM. (**b**) Intensity field distribution of the FM under plane incidence. (**c**) Pressure distribution of the FT block. (**d**) Transmitted pressure (normalized by the maximum value) of the FT block together with the analytical result.

The desired transmission coefficient of the FM can be expressed as

5 $${T}_{f}(y)=\exp [i\frac{2\pi }{\lambda }(\sqrt{{y}^{2}+{L}^{2}}-L)]$$

where *λ* is wavelength in the air at the operating frequency, *L* = 4*λ* is the focal length. It should be noted that the focal length of the FM can be designed as a desired value^30^. Figure 3a shows the discrete transmission coefficient (circles) provided by the designed FM and the desirable continuous transmission coefficient (lines) described by Eq. (5). As can be seen, the discrete transmission phase shows a hyperboloidal profile and is in good agreement with the required transmission phase. We carry out a numerical simulation to verify the focusing functionality of the FM. For a plane incident signal, Fig. 3b illustrates the simulated intensity field distribution of the fabricated FM, where the intensity field is calculating as the square of the amplitudes of the acoustic pressure field. It is observed that the transmitted energies are focused at the focus point, which demonstrates that the fabricated FM has excellent focusing effect. The constructed FM and its two neighboring blocks of air form the FT block, and we carry out a numerical simulation to confirm its functionality of the Fourier transform. Figure 3c shows the pressure distribution of the FT block with the incident signal being *P*_i_(*y*) = exp(−50*y*^2^). It can be seen that the transmitted pressure is strong in the central region, and tis symmetric about *y* = 0. Figure 3d shows the corresponding normalized transmitted pressure (circles) of the FT block. The transmitted pressure presents an axial symmetric form, and a peak of pressure is occurred at *y* = 0, which agrees well with the analytical result (red line). Therefore, we realize the FT block with excellent functionality based on the constructed FM. It is mentioned that the spatial frequency *k*_*y*_ and the transverse variable *y* are related to each other via the relation of *k*_*y*_ = *εy* with $\varepsilon =-\,\frac{2{\rm{\pi }}}{{\lambda }_{0}L}$^28^.

### Two exampled acoustic ODE solvers

The desired transmission coefficient of SFM is associated with the transfer function *H*(*k*_*y*_). Here, we design two SFMs to solve ODEs for an incident signal *P*_i_(*y*) [ = *f*_1_(*y*) = 500*y*^2^exp(−50*y*^2^) − 55exp(−50*y*^2^)]. The first SFM is designed for solving a second-order ODE (*n* = 2) such as *f*_1_(*y*) = 0.05 *g*″(*y*) − 50 *g*(*y*), whose analytical solution follows as *g*(*y*) = exp(−50*y*^2^). According to Eq. (3), the transfer function takes the form *H*(*k*_*y*_) = [0.05(*ik*_*y*_)^2^ − 50]^−1^, resulting in a required transmission coefficient of the SFM, i.e., *T*_s_(*y*) ∝ *H*(*k*_*y*_). Since a low transmission coefficient causes a high consumption, we assume the maximum magnitude of the required transmission coefficient to be unity. Then the required transmission coefficient can be expressed as

6 $${T}_{s}(y)=\frac{50}{0.05{(i\varepsilon y)}^{2}-50}$$

**Figure 4:**
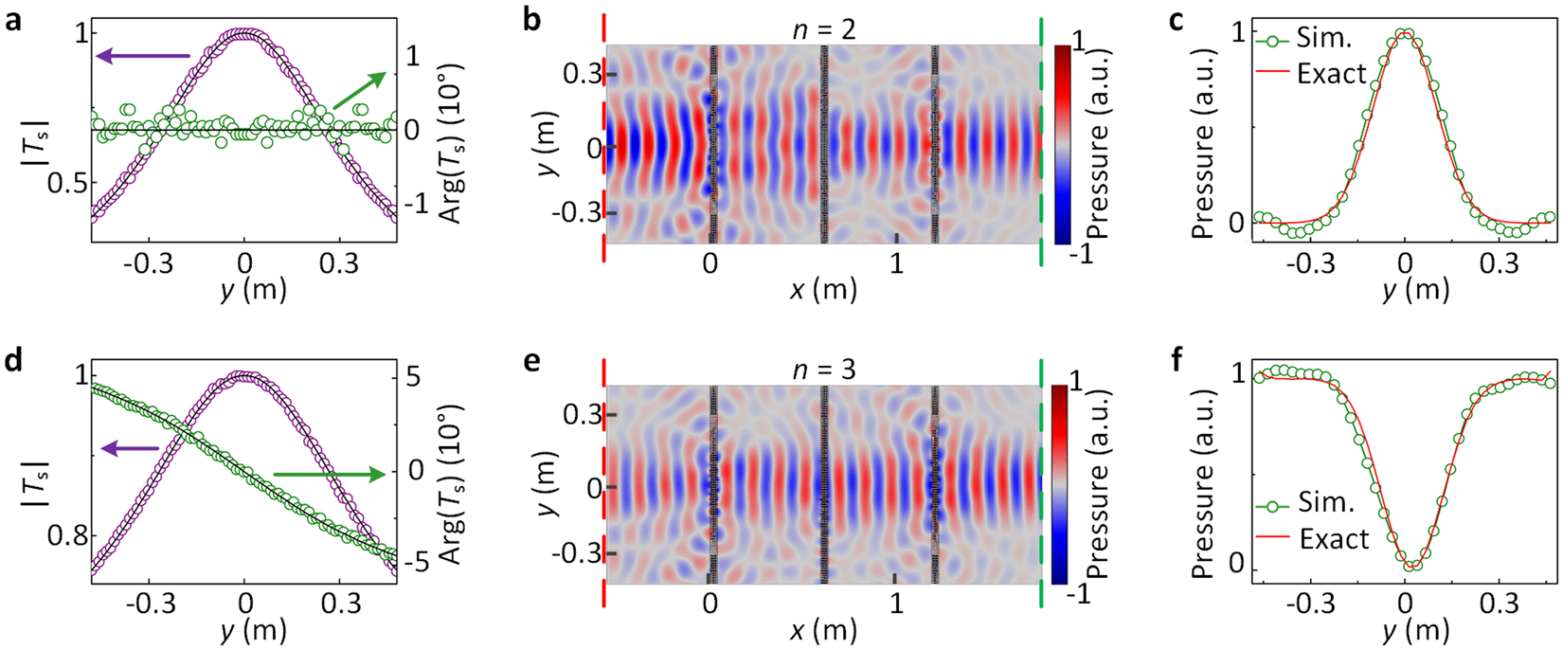
Simulation results of the AAC system for *P*_i_(*y*) = *f*_1_(*y*). Simulations of two AAC systems designed for solving [(**a**–**c**)] a second-order ODE and [(**d**–**f**)] a third-order ODE, respectively. [(**a**,**d**)] Designed discrete transmission coefficient (circles) and the required ideal transmission coefficient (lines) of the two SFMs. [(**b**,**e**)] Pressure field distributions of the two AAC systems. [(**c**,**f**)] Normalized transmitted pressures of the two AAC systems together with corresponding analytical solutions.

To implement the transmission coefficient in Eq. (6), we construct the SFM, whose discrete transmission coefficient is shown by circles in Fig. 4a. It is found that the transmission amplitude is in a parabolic-like profile and the transmission phase fluctuates around 0°, which agrees well with the required transmission coefficient (black lines). By inserting the designed SFM into the middle of two fabricated FT blocks, Fig. 4b shows the pressure distribution of the AAC system, where the signal *P*_i_(*y*) is incident on the AAC system at *x* = −0.5488 m (red dash line), and the transmitted signal *P*_t_(*y*) is obtained at *x* = 1.7664 m (green dash line). At the output plane, the pressure is strong around *y* = 0 and symmetric about *y* = 0. Figure 4c shows the transmitted pressure, which presents a Gauss profile peaking at *y* = 0 and agrees with the analytical solution g(*y*). Thus, the AAC system can solve the second-order ODE.

The second SFM is designed to solve a third-order ODE (*n* = 3) such as *f*_1_(*y*) = −0.005 *g*′′′(*y*) − 0.2 *g*′′(*y*) + 10 *g*′(*y*) + 400 *g*(*y*). The transfer function of the ODE is given by *H*(*k*_*y*_) = [−0.005(*ik*_*y*_)^3^ − 0.2(*ik*_*y*_)^2^ + 10*ik*_*y*_ + 400]^−1^, and the corresponding required transmission coefficient can be deduced to

7 $${T}_{s}(y)=\frac{400}{-0.005{(i\varepsilon y)}^{3}-0.2{(i\varepsilon y)}^{2}+10i\varepsilon y+400}$$

Figure 4d shows the discrete transmission coefficient (circles) provided by the designed SFM. The transmission amplitude presents an analogous parabolic profile, and its phase decreases monotonically with increasing *y*. The designed transmission coefficient is consistent with the required transmission coefficient (black lines). Figure 4e shows the pressure distribution of the AAC system. It can be observed that the pressure is relatively strong around *y* = 0 at the output plane. Figure 4f shows the transmitted pressure of the system, which presents a valley-like form and accords with the analytical solution. The simulation results confirm that the designed AAC system could solve the third-order ODE.

**Figure 5:**
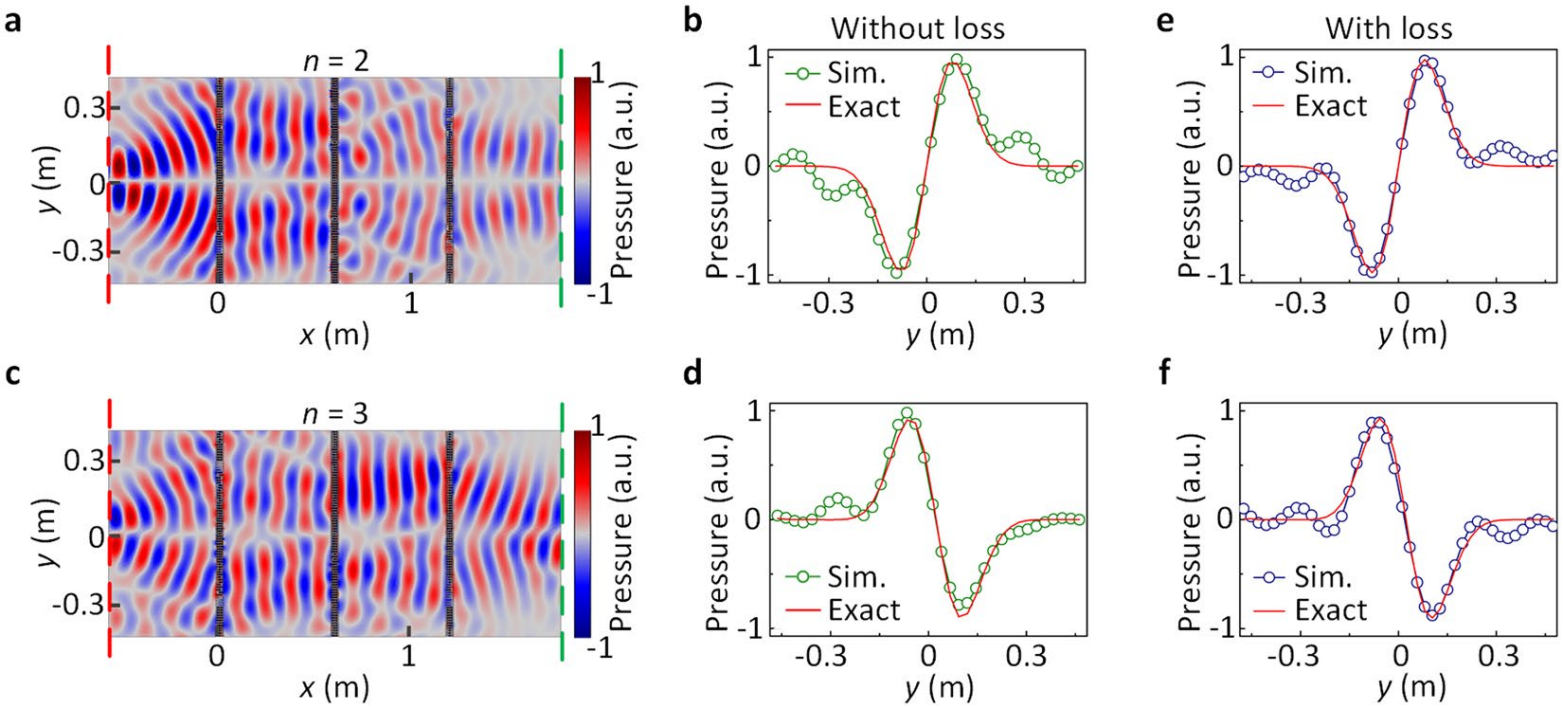
Simulation results of the AAC system for *P*_i_(*y*) = *f*_2_(*y*). The SFMs used to solve the second-order and the third-order ODEs are same as their counterparts in Fig. 4. Pressure distributions of the systems for solving (**a**) the second-order and (**c**) the third-order ODEs. Corresponding normalized transmitted pressures at the output planes for solving (**b**) the second-order and (**d**) the third-order ODEs together with the analytical solutions. In thermoviscous case, the transmitted pressures for solving (**e**) the second-order and (**f**) the third-order ODEs.

The designed AAC system is feasible for arbitrary incident signals at the designed operating frequency 2500 Hz (see details in Supplementary information). For example, we change the incident signal in the simulations of Fig. 4 into *P*_i_(*y*) [=*f*_2_(*y*) = 1280*y*^3^exp(−80*y*^2^) − 74*y*exp(−80*y*^2^)]. The SFMs are the same for solving the second-order and the third-order ODEs with their counterparts. Figures 5a,c show the pressure distributions of the AAC system to solve the second-order and the third-order ODEs, respectively. Figures 5b,d depict the corresponding transmitted pressures. For the system to solve the second-order ODE, the pressure is weak around *y* = 0, where a π-phase difference is observed (Fig. 5a). On the other hand, the transmitted pressure approaches zero at *y* = 0, and appears a valley (peak) in the region of *y* < 0 (*y* > 0), as shown in Fig. 5b, which agree with the analytical solution g(*y*) = *y*exp(−80*y*^2^). For the system to solve the third-order ODE, the pressure is weak around *y* = 0 (Fig. 5c), and the transmitted pressure presents a peak (valley) profile in the region of *y* < 0 (*y* > 0) (Fig. 5d). The simulated results agree with the analytical solution. Hence, the designed AAC systems for solving the ODEs are feasible for arbitrary incident signal.

Although the layered labyrinthine unit is designed with lossless assumption, the performance is also confirmed in simulation by considering visco-thermal loss since it is the inherent loss of the structure. We insert the AAC systems (Fig. 5a,c) into the COMSOL Multiphyiscs and use the thermoviscous acoustics model instead of the ideal loss-free model. For the input signal *P*_i_(*y*) = *f*_2_(*y*), Fig. 5e,f show the transmitted signals of AAC systems for solving the second-order and the third-order ODEs, respectively. As can be seen, the simulated results in Fig. 5e,f accord with the analytical solutions, indicating that the performance of the designed AAC system will not be severely influenced when loss is considered. Obviously, the losses would impact more or less on the amplitude and phase modulation performances. However, the operating frequency here (2500 Hz) is not high and the labyrinthine units are not resonate-based^24^. Therefore, the visco-thermal losses of the units are weak, and the functionality of the AAC system is not very sensitive to the weak visco-thermal losses.

### Geometrical width of incident signal

In the simulation results (Figs 4 and 5), some discrepancies can be observed. The reasons for the discrepancies are described in Supplementary information. To improve the functionality of the AAC system, we restrict the geometrical width of incident signal by two conditions. On the one hand, the ideal FT on the incident signal *P*_i_(*y*) is defined as

8 $${{\rm{FT}}}_{{\rm{ideal}}}[{P}_{i}(y)]={\int }_{-\infty }^{\infty }{P}_{i}(y)\exp (\,-\,i{k}_{y}y)dy$$

For the FT block based on FM, the integration area is limited by the width *W*, and the FT on incident signal *P*_i_(*y*) is described by

9 $${\rm{FT}}[{P}_{i}(y)]={\int }_{-W/2}^{W/2}{P}_{i}(y)\exp (\,-\,i{k}_{y}y)dy$$

which should be equal to FT_ideal_[*P*_i_(*y*)] to promise the FT block with good functionality. Comparing Eq. (9) to Eq. (8), the incident signal *P*_i_(*y*) must be integrable in the region (−∞, +∞), and the energy should be concentrated in the region of (−*W*/2, *W*/2), which is the first condition.

On the other hand, the FMs and SFM are low-pass filters in analogy with the optical lenses, whose cutoff frequencies are |*k*_*yc*_| = *εW*/2. The Fourier spectrum of the incident signal is ${S}_{0}({k}_{y})=|{\rm{FT}}[{P}_{i}(y)]|$^31^, whose all frequency contents contribute to the transmitted signal. Due to the diffraction, the Fourier spectrum *S*_0_(*k*_*y*_) is variable in air, resulting in some high frequency contents. The FMs and SFM can complete eliminate the contents beyond *k*_*yc*_, leading to some discrepancies in Figs 4 and 5. Though the high frequency contents are hard to avoid in the propagation, they can be reduced by defining the geometrical width of incident signal. Here, we only consider the first FM, because a majority of high frequency contents are filtered by the first FM. The Fourier spectrum at the filtering plane (*x* = 0) is^32^

10 $$S({k}_{y})=|{\rm{FT}}[{P}_{i}(y)]\exp (\frac{iL{\lambda }_{0}}{4\pi }{k}_{y}^{2})|$$

**Figure 6:**
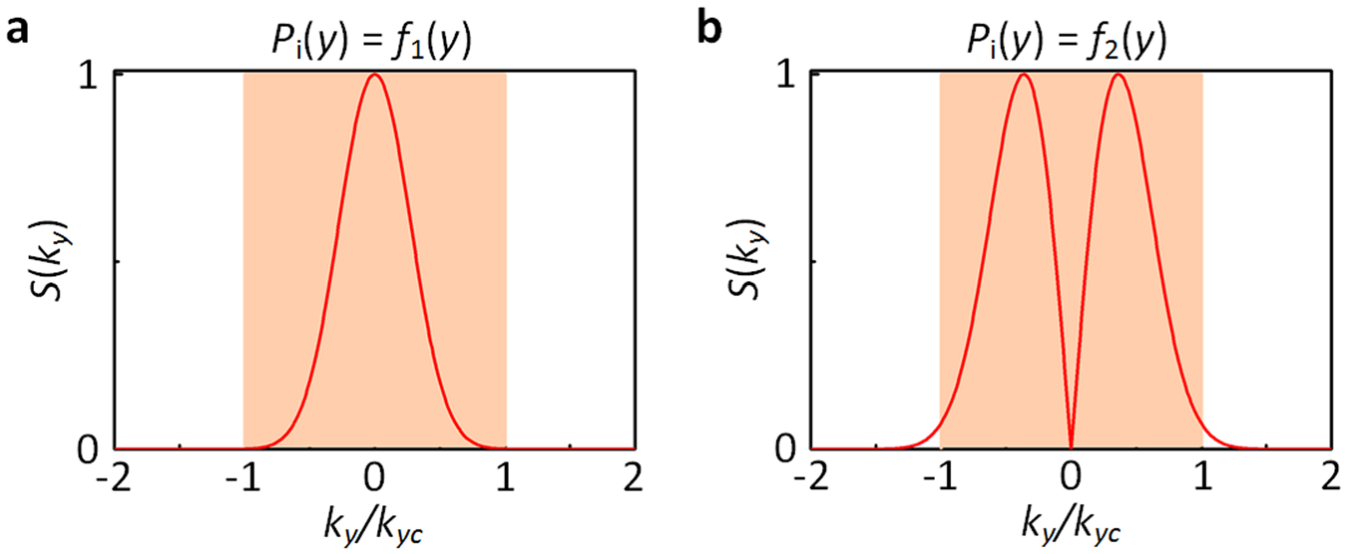
Fourier spectral analysis on incident signals. The Fourier spectrums for (**a**) *P*_i_(*y*) = *f*_1_(*y*) and (**b**) *P*_i_(*y*) = *f*_2_(*y*). The yellow region denotes the passing band, in which the frequency contents can propagate through.

By analyzing the Fourier spectrum *S*(*k*_*y*_), we can explain why the results for *P*_i_(*y*) = *f*_1_(*y*) (Fig. 4) are better than those for *P*_i_(*y*) = *f*_2_(*y*) (Fig. 5). Figure 6a,b show the Fourier spectrums *S*(*k*_*y*_) for *P*_i_(*y*) = *f*_1_(*y*) and *P*_i_(*y*) = *f*_2_(*y*), respectively. As shown in Fig. 6a, all frequency contents are in the passing band (yellow region), indicating that no content is filtered by the first FM. In this case, few discrepancies between the simulation and exact results can be observed (Fig. 4c,f). On the contrary, some frequency contents in Fig. 6b beyond |*k*_*yc*_|, which will be filtered by the first FM, causing some discrepancies between the simulation and exact results (Fig. 5b,d). Therefore, the distribution of Fourier spectrum *S*(*k*_*y*_) is critical to the performance of the AAC system. To improve the accuracy of the AAC system, the ratio of low frequency contents $({\int }_{-{k}_{yc}}^{{k}_{yc}}S({k}_{y})d{k}_{y})$ to all frequency contents $({\int }_{-\infty }^{\infty }S({k}_{y})d{k}_{y})$ should be above 98%, which is the second condition.

## Discussion

In this work, we have developed an acoustic AAC system to solve the *n*th-order ODEs. The proposed system consists of two FMs and one SFM, which are constructed by layered labyrinthine metamaterials. The FM forms the acoustic FT block, while the SFM exhibits a complex transmission coefficient to mimic the desired transfer function of the ODE. We have demonstrated that the AAC system is capable to solve the *n*th-order ODEs for arbitrary incident functions. Due to the inherent merits of metasurfaces, such as low power consumption and small size, the ODE solver features fixed geometry, high efficiency and broad adjustability. The proposed system may provide an effect way to solve ODEs in acoustic domain, which could be a functional component for acoustic communication, analog computing and signal processing.

## Methods

### Simulations

Throughout the paper, Finite Element Method based on commercial software COMSOL Multiphysics^TM^ 5.2a is employed for the simulations. The labyrinthine unit applied in the simulations is stiff curvature (sound hard boundaries). To calculate the transmission coefficient of the labyrinthine unit with different spiral radians (Fig. 2b and c), the plane wave radiation boundary condition is imposed on the incident and transmitted boundaries, and the periodic boundary condition are employed in the *y* direction. For Figs. 3–5, plane wave radiation boundaries are imposed on the outer boundaries of simulated domain to eliminate the interference from reflected wave. The designed FM and SFM with realistic structures will reflect some signals, which results in the transmitted signals containing the desired diffraction signals *P*_t_(*y*) and some noise signals. To decrease the reflection between the FM and SFM, their gap should be a large value and hence we choose the focal length as *L* = 4*λ* in this paper. The large *L*-value promises a balance between the system size and the reduced scattered signals (see Supplementary information).

### Data availability

The datasets generated during and/or analyzed during the current study are available from the corresponding author on reasonable request.

## Electronic supplementary material

Supplementary Information
