# Supplementary material for: Acoustic analog computing system based on labyrinthine metasurfaces
Source: Sci Rep. 2018 Jul 4;8:10103. doi: 10.1038/s41598-018-27741-2 (PMC6031674; doi:10.1038/s41598-018-27741-2)
Supplement: Supplementary file 1 — Supplementary Information [file 41598_2018_27741_MOESM1_ESM.pdf]

Supplementary Information for

Acoustic analog computing system based on labyrinthine metasurfaces

Shuyu zuo[1], Qi Wei[1,2], Ye Tian[1], Ying Cheng[1,3] & Xiaojun Liu[1,3]

[1]Key Laboratory of Modern Acoustics, Department of Physics and Collaborative Innovation Center of Advanced Microstructures, Nanjing University, Nanjing 210093, China.

[2]Jiangsu Key Laboratory on Opto-Electronic Technology, School of Physics and Technology, Nanjing Normal University, Nanjing 210023, China.

[3]State Key Laboratory of Acoustics, Institute of Acoustics, Chinese Academy of Sciences, Beijing 100190, China.

Correspondence and requests for materials should be addressed to Q.W. (email: weiqi@njnu.edu.cn) or X.L. (email: liuxiaojun@nju.edu.cn)

Supplementary Note: The bandwidth analysis

The AAC system only works at the designed operating frequency 2500 Hz, because the FM and SFM are designed at operating frequency. When the frequency changes, the transmission coefficients of the designed FM and SFM (circles in Figs. 3a, 4a and 4d) will deviate from the desired transmission coefficients, which may result in a discrepancy between the transmitted signal and the theoretical solution. For the incident signal $P_i(y) = f_1(y)$, Supplementary Fig. S1a shows the root-mean-square errors (RMSEs) between the simulated results and the analytical solutions, where the AAC system is same as that shown in Fig. 4b. As can be seen, the RMSEs are in the lowest valley when the frequency is 2500 Hz, because the AAC system has the best functionality at the operating frequency. For comparison, Supplementary Figs. S1b and S1c show the transmitted signals of the AAC system at 2500 Hz and 2420 Hz, respectively. As can be seen, the simulated result at operating frequency is more in agreement with the analytical solution. To promise the accuracy of the simulated results, the AAC system is considered as a single frequency ODE solver.

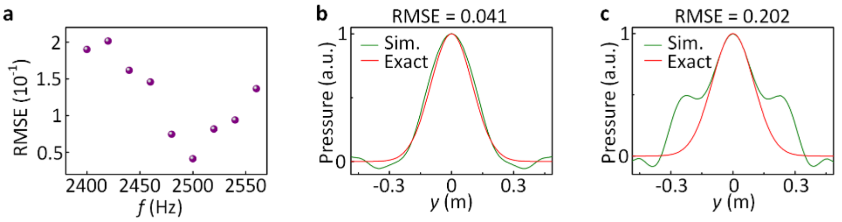

Supplementary Figure S1. Bandwidth analysis. (**a**) The RMSEs between the expect solutions and simulated transmitted signals, where $\mathrm{RMSE} = \sqrt{\sum_{n=1}^{N}\left[P_t(y_n) - g(y_n)\right]^2 / (N-1)}$, and N = 295 is the sampling number along y direction. Normalized transmitted pressures of the AAC system for (**b**) 2500 Hz and (**c**) 2420 Hz, respectively.

Supplementary Note: The reasons for simulation discrepancies

The AAC system can be considered as a filter, prohibiting the passage of signal whose transverse variable is above $|W/2|$. The expected solution described by $P_t(y)$ = FT{$T_s(y)$FT[$P_i(y)$]} contains all diffraction signals of the input signals. When some signals are filtered, the transmitted signals will not strictly equal to the expected solution. The filter phenomena are plotted in Supplementary Fig. S2, where the AAC system is surrounded by blue rectangle and the remaining areas are air. The FM and SFM with ideal parameters features no reflection, promising the pressure distribution is composed by diffraction signals. When the diffraction signals exceed the blue rectangle, they will not contribute to the transmitted signals of the AAC system, which will be filtered.

The unsuitable input signals and the SFM with asymmetry can cause the filter phenomena. On the one hand, for some input signals, the diffraction signals in the first air region could exceed the blue rectangle. For example, when the input signals show $P_i(y) = f_1(y)$, few signal is filtered at the first air region, as shown in Supplementary Figs. S2a and S2b. Conversely, when the input signals show $P_i(y) = f_2(y)$, some signals are filtered at the first air region, which are circled by red lines in Supplementary Figs. S2c and S2d. On the other hand, the signals transmitted from the SFM with asymmetry deviate from x-direction, whose direction is shown as black arrows in Supplementary Figs. S2b and S2d. The diffraction signals propagating along the black arrows will exceed the blue rectangle, and those signals (denoted by the black circle) will be filtered.

Besides, unlike the ideal case, the designed FM and SFM with realistic structures will reflect some signals rather than absorb them. The reflection between the FM and SFM also

affects the transmitted signals of the AAC system. For example, Supplementary Fig. S2e shows the sketch of reflection between the SFM and FM. In this figure, the blue arrow denotes the desired propagation of the incident signals and its corresponding transmitted signals $P_t(y) \propto g(y)$, and the purple arrows denote the propagation of noise signals caused by the reflection. To descrese the reflection between the FM and SFM, their gap should be large as possible.

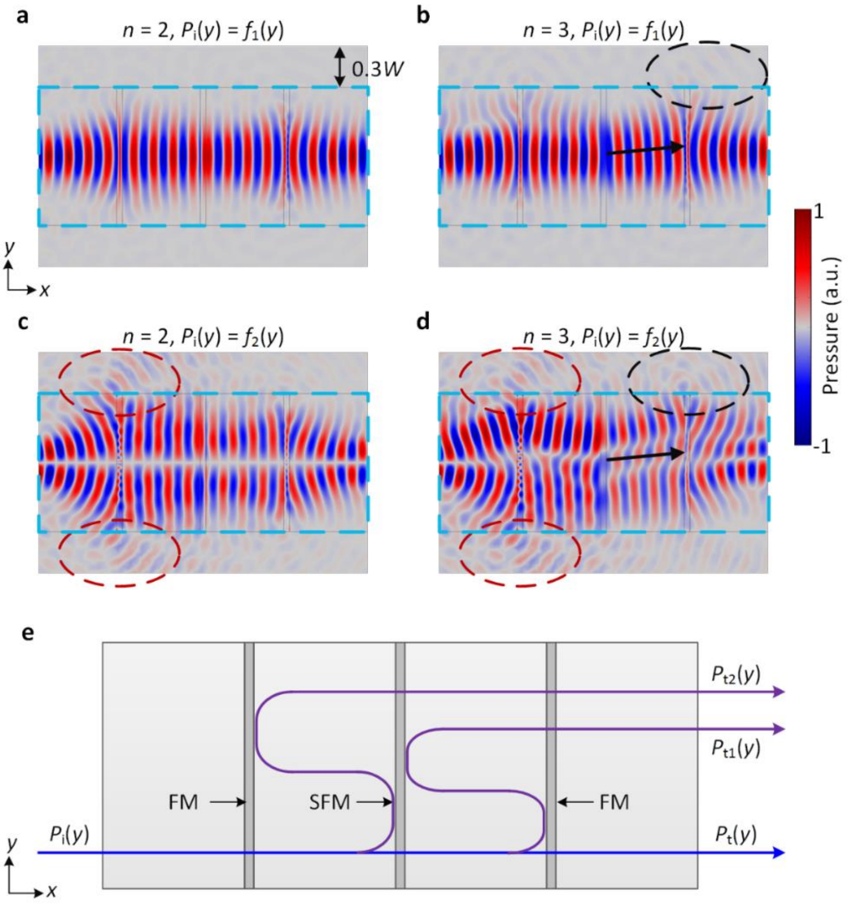

Supplementary Figure S2. The discrepancies caused by filter and reflection. [(**a**)-(**d**)] The AAC system is denoted by blue rectangle, and the filtered signals are circled by the lines. The air layer with a width $0.3W$ is placing at the top and bottom of the AAC system. (**e**) The evolutions of reflections between the FM and SFM.

Supplementary Note: The design of the focal length

A focusing lens can perform spatial FT and its focal length can be designed as a desired value. For example, Supplementary Fig. S3a shows the designed transmission coefficient (circles) of the FM with a focal length of $L = 2\lambda$. Supplementary Fig. S3b shows the pressure distribution of the AAC system, where the focal length is $L = 2\lambda$ and the input signal is $P_i(y) = f_1(y)$, and the SFM is same as that shown in Fig. 4b. Supplementary Fig. S3c shows the transmitted pressure of the AAC system, whose performance is not as good as the AAC system with $L = 4\lambda$ (Fig. 4c). This is because that the designed FM and SFM with realistic structures will reflect some signals, resulting in the transmitted signals containing the desired signals (red arrow in Supplementary Fig. S3b) and some noise signals (black arrows in Supplementary Fig. S3b). To decrease the reflection between the FM and SFM, their gap should be a large value and hence we choose the focal length as $L = 4\lambda$ in this paper.

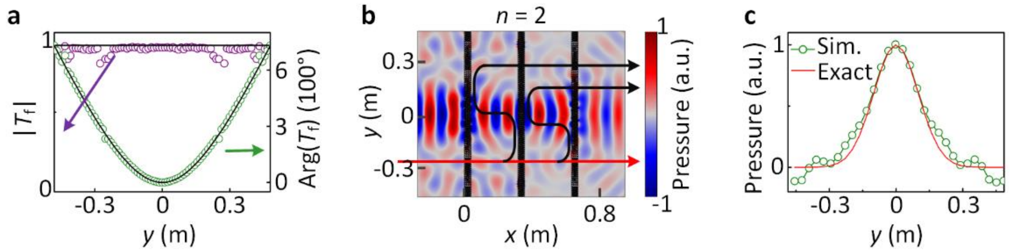

Supplementary Figure S3. Simulation results of the AAC system with the focal length being 2λ. (**a**) Designed discrete transmission coefficient (circles) and the required ideal transmission coefficient (lines) of FM. (**b**) Pressure field distribution of the AAC system. The signal $P_i(y)$ is incident on the AAC system at $x = -0.2744$ m, and the transmitted signal $P_t(y)$ is obtained at $x = 0.8472$ m. (**c**) Normalized transmitted pressure of the AAC systems together with corresponding analytical solutions.
